# The association of six polymorphisms of five genes involved in three steps of nucleotide excision repair pathways with hepatocellular cancer risk

**DOI:** 10.18632/oncotarget.7952

**Published:** 2016-03-07

**Authors:** Bengang Wang, Qian Xu, Huai-wei Yang, Li-ping Sun, Yuan Yuan

**Affiliations:** ^1^ Tumor Etiology and Screening Department of Cancer Institute and General Surgery, The First Affiliated Hospital of China Medical University, and Key Laboratory of Cancer Etiology and Prevention (China Medical University), Liaoning Provincial Education Department, Shenyang 110001, China; ^2^ Hepatobiliary Surgery Department of General Surgery Institute, The First Affiliated Hospital of China Medical University, Shenyang 110001, China

**Keywords:** nucleotide excision repair, single nucleotide polymorphism, hepatocellular cancer, interaction

## Abstract

**Background:**

Hundreds of single nucleotide polymorphisms (SNPs) of the genes encoding nucleotide excision repair (NER) proteins are involved in every step of the DNA recognition–unwinding–incision process, which may affect cancer risk. However, only a limited number of studies have examined the association of NER SNPs with hepatocellular cancer (HCC) risk.

**Results:**

In screening stage, single-locus analysis showed that six SNPs in five genes were associated with HCC risk, including three risk SNPs (*XPA* rs10817938, *XPC* rs1870134 and *ERCC2* rs238417) and three protective SNPs (*ERCC1* rs2298881 and rs3212961, and *ERCC5* rs873601). In verification stage, only *XPC* rs1870134 was verified to be associated with HCC risk (*P* = 4.7 × 10^−4^). Furthermore, multivariate logistic regression and MDR analysis consistently revealed a gene–gene interaction among ERCC1 rs2298881 and *XPC* rs1870134 SNPs associated with HCC risk (*P*_interaction_ = 0.023). When analyzing the effect of the positive SNP on the mRNA expression, we found *XPC* rs1870134 GG genotype which was associated with an increased HCC risk showed lower *XPC* mRNA expression.

**Methods:**

This study designed as “screening-verification” experiments and included a total of 1472 participants (570 HCC patients vs. 902 controls). We explored 39 SNPs in eight genes involved in NER Pathways, including *XPA, XPC, DDB2, ERCC3, ERCC2, ERCC1, ERCC4* and *ERCC5*, using Sequenom MassARRAY and KASPar platform. Eighty-six cases of HCC and the neighboring noncancerous tissues were subjected to the measurement of mRNA expression level of the promising gene.

**Conclusions:**

*XPC* promoter rs1870134 SNP and SNP-SNP interaction were associated with HCC risk.

## INTRODUCTION

Hepatocellular cancer (HCC) is the sixth most common type of cancer and the third most frequent cause of cancer death worldwide [1, 2]. The incidence of HCC is associated with environmental and hereditary factors, therefore the risk of developing disease varies between patients. To date, several single nucleotide polymorphisms (SNPs) in some genes involved in oxidative stress, metabolism and inflammation pathways, have been proved to be associated with HCC risk [3]. These SNPs have great significance for the selection of individuals who benefit from specific preventative measures [3].

DNA repair systems include nucleotide excision repair (NER), base excision repair, mismatch repair, and double-strand break repair [4]. Among these repair systems, the NER system is most frequently associated with cancer [5]. Ishikawa et al. previously reported that the DNA repair system, especially the NER pathway, played a vital role in protection against human cancer [6].

The NER pathway is composed of DNA recognition-related proteins including *XPA*, *XPC*, and *DDB2*; DNA unwinding-related proteins such as *XPB* (*ERCC3*) and *XPD* (*ERCC2*); and DNA incision-related proteins, for instance *ERCC1*, *XPF* (*ERCC4*), and *XPG* (*ERCC5*). Hundreds of SNP variants of the genes encoding these NER proteins are involved in every step of the DNA recognition–unwinding–incision process, which may increase or decrease protein expression and function [7, 8]. However, only a limited number of studies have examined the association of NER SNPs with HCC risk, although a few studies focused on single exon SNPs such as *XRCC1* Arg399Gln, *XRCC3* Thr241Met, and *XPD* Lys751Gln have been reported [9–12]. And there was a meta-analysis investigating the association of NER SNPs with risks of several kinds of cancers [13] without hepatocellular cancer, which maybe because that few studies were performed about the association of NER SNPs with HCC risk. Thus, a systematic and comprehensive evaluation of the relationship between these SNPs and HCC risk are urgently required, which could provide a comprehensive understanding of the implications of NER biological pathways involved in hepatocarcinogenesis, as well as screening the most significant functional SNP variants and potential biomarkers for predicting HCC risk.

In the present study, we adopted candidate gene association study strategy with selected 39 potentially functional tag SNPs (tagSNPs) in eight genes involved in NER pathways: *XPA*, *XPC*, *DDB2*, *ERCC1*, *ERCC2*, *ERCC3*, *ERCC4* and *ERCC5*. We determined whether these genes were associated with HCC in the second verification stage, and for the promising SNPs we investigated the effects of the SNPs on the mRNA expression of the corresponding genes. We aimed to identify predictive biomarkers for HCC risk and tried to establish an experimental basis to improve understanding of the etiology and the mechanism of HCC.

## RESULTS

### The association of SNPs in NER pathway genes with hepatocellular cancer risk

The demographic characteristics of HCC and control subjects are shown in Supplementary Table S1. All polymorphism genotype distributions in cases and controls are shown in Supplementary Table S2, including 39 SNPs in eight genes (recognition-related: *XPA* rs10817938, rs3176629, rs3176658, rs2808668; *XPC* rs2607775, rs1870134, rs2228000, rs2228001, rs2470352; *DDB2* rs2029298, rs830083, rs3781619, rs326222; unwinding-related: *ERCC3* rs4150441, rs4150448, rs4150506; *ERCC2* rs238406, rs50871, rs50872, rs238417, rs1052555, rs13181; incision-related: *ERCC1* rs2298881, rs11615, rs3212955, rs3212961, rs3212986, rs735482; *ERCC4* rs254942, rs1799801, rs2276464; and *ERCC5* rs2094258, rs751402, rs2296147, rs1047768, rs4150291, rs2228959, rs4150383, and rs873601). Among them, most SNPs conformed to Hardy-Weinberg equilibrium (HWE) including SNPs in stage 1 and 2, except for DDB2 rs326222 (*P*_HWE_ = 0.022), ERCC2 rs50871 (*P*_HWE_ = 5 × 10^−7^), ERCC5 rs751402 (*P*_HWE_ = 0.018) and rs2228959 (*P*_HWE_ = 0.013) in stage 1. Therefore these four SNPs were discarded in further association analysis.

Among these 39 SNPs, six SNPs in five genes were associated with HCC risk in stage 1, including three risk SNPs (*XPA* rs10817938, *XPC* rs1870134 and *ERCC2* rs238417) and three protective SNPs (*ERCC1* rs2298881 and rs3212961, and *ERCC5* rs873601). We further analyzed these promising SNPs and found that the *XPA* rs10817938 variant CC genotype showed an increased risk of HCC (odds ratio [OR] = 2.52 and 2.66, respectively; Table 1) when compared with TT wild-type and TT + TC genotype. The *ERCC2* rs238417 variant CC genotype also showed an increased risk (OR = 1.77 and 1.33, respectively) under the allelic model. And the *XPC* rs1870134 variant GG + GC genotype showed an increased risk for HCC (OR = 2.78) when compared with CC genotype. By contrast, the *ERCC5* rs873601 variant AA genotype had a decreased risk for HCC (OR = 0.58 and 0.59, respectively) when compared with GG wild-type and under the recessive model. Two positive SNPs were identified in *ERCC1*, rs2298881 and rs3212961, which were associated with a decreased HCC risk (OR = 0.64 and 0.60, respectively, Table 1) when the heterozygote was compared with the wild-type.

**Table 1:** The association of promising SNPs of NER pathway genes with hepatocellular cancer risk

Gene	SNP	Genotype	Stage 1				Stage 2				Meta	
			Cases (%)	Controls (%)	*P*^a^	OR (95%CI)	Cases (%)	Controls (%)	*P*^a^	OR(95%CI)	*P*^a^	OR (95%CI)
			*n* = 169	*n* = 501			*n* = 401	*n* = 401				
**Recognition-related**												
XPA	rs10817938						*n* = 353	*n* = 379				
		TT	105 (62.1)	311 (62.1)		1(Ref.)	208 (58.9)	241 (63.6)		1 (Ref.)		
		TC	51 (30.2)	172 (34.3)	0.525	0.88 (0.60–1.30)	129 (36.5)	124 (32.7)	0.263	1.19 (0.88–1.63)	0.537	1.08 (0.85–1.36)
		CC	13 (7.7)	15 (3.0)	**0.020**	**2.52 (1.16–5.49)**	16 (4.5)	14 (3.5)	0.098	2.04 (0.88–4.73)	0.038	1.76 (1.03–3.01)
		CC+CT vs. TT			0.956	1.01 (0.70–1.45)			0.146	1.25 (0.93–1.69)	0.260	1.14 (0.91–1.42)
		CC vs. TT+TC			**0.012**	**2.66 (1.24–5.74)**			0.136	1.88 (0.82–4.33)	0.045	1.72 (1.01–2.91)
XPC	rs1870134						*n* = 359	*n* = 381				
		GG	88 (52.1)	257 (51.3)		1 (Ref.)	230 (64.1)	197 (51.7)		1 (Ref.)		
		GC	76 (45.0)	204 (40.7)	0.654	1.09 (0.76–1.56)	114 (31.8)	160 (42.0)	**0.001**	**0.60 (0.44–0.82)**	0.009	0.74 (0.59–0.93)
		CC	5 (3.0)	39 (7.8)	**0.048**	**0.39 (0.14–0.99)**	15 (4.2)	24 (6.3)	0.067	0.53 (0.27–1.05)	0.002	0.44 (0.26–0.75)
		GG vs. CC+GC			0.869	1.03 (1.01–1.47)			**4.7 × 10^−4^**	**1.67 (1.25–2.27)**	**0.001**	**1.45 (1.15–1.79)**
		GG+GC vs. CC			**0.034**	**2.78 (1.09–7.14)**			0.214	1.52 (0.78–2.94)	0.010	1.96 (0.56–3.33)
**Unwinding-related**												
XPD(ERCC2)	rs238417						*n* = 345	*n* = 372				
		GG	39 (23.1)	149 (29.7)		1 (Ref.)	127 (36.8)	148 (39.8)		1 (Ref.)		
		GC	87 (51.5)	248 (49.5)	0.191	1.33 (0.87–2.05)	158 (45.8)	155 (41.7)	0.249	1.21 (0.87–1.68)	0.491	1.09 (0.85–1.40)
		CC	43 (25.4)	90 (18.0)	**0.028**	**1.77 (1.06–2.95)**	60 ((17.4)	69 (18.5)	0.796	1.06 (0.69–1.62)	0.330	1.17 (0.85–1.60)
		CC+GC vs. GG			0.068	1.46 (0.97–2.20)			0.310	1.17 (0.86–1.59)	0.349	1.12 (0.86–1.41)
		CC vs. GG+GC			0.056	1.50 (0.99–2.27)			0.794	0.95 (0.65–1.40)	0.437	1.12 (0.85–1.47)
**Incision-related**												
ERCC1	rs2298881						*n* = 329	*n* = 388				
		CC	79 (46.7)	194 (38.7)		1 (Ref.)	104 (31.6)	145 (37.4)		1 (Ref.)		
		CA	58 (34.3)	224 (44.7)	**0.027**	**0.64 (0.44–0.95)**	163 (49.5)	191 (49.2)	0.302	1.19 (0.86–1.65)	0.953	0.99 (0.78–1.27)
		AA	32 (18.9)	81 (16.2)	0.929	0.98 (0.60–1.59)	62 (18.8)	52 (13.4)	0.020	1.71 (1.09–2.68)	0.096	1.31 (0.95–1.81)
		CA+AA vs. CC			0.076	0.73 (0.51–1.03)			0.100	1.30 (0.95–1.77)	0.563	1.07 (0.85–1.34)
		AA vs. CC+CA			0.427	1.20 (0.76–1.89)			0.036	1.54 (1.03–2.31)	0.061	1.32 (0.99–1.77)
	rs3212961						*n* = 367	*n* = 388				
		CC	64 (37.9)	143 (28.5)		1 (Ref.)	124 (33.8)	119 (30.7)		1 (Ref.)		
		CA	66 (39.1)	251 (50.1)	**0.012**	**0.60 (0.40–0.89)**	158 (43.1)	188 (48.5)	0.211	0.81 (0.58–1.13)	0.008	0.72 (0.56–0.92)
		AA	39 (23.1)	106 (21.2)	0.422	0.83 (0.51–1.32)	85 (23.2)	81 (20.9)	0.889	1.03 (0.69–1.53)	0.600	0.92 (0.69–1.24)
		CA+AA vs. CC			0.028	0.66 (0.46–0.96)			0.392	0.88 (0.64–1.19)	0.032	0.78 (0.62–0.98)
		AA vs. CC+CA			0.612	1.11 (0.73–1.69)			0.399	1.16 (0.82–1.64)	0.353	1.13 (0.87–1.46)
XPG(ERCC5)	rs873601						*n* = 369	*n* = 394				
		GG	54 (32.0)	142 (28.3)		1 (Ref.)	109 (29.5)	129 (32.7)		1 (Ref.)		
		GA	87 (51.5)	231 (46.1)	0.966	0.99 (0.66–1.48)	184 (49.9)	177 (44.9)	0.223	1.23 (0.88–1.71)	0.414	1.11 (0.87–1.42)
		AA	28 (16.6)	126 (25.1)	**0.041**	**0.58 (0.35–0.98)**	76 (20.6)	88 (22.3)	0.897	1.03 (0.69–1.53)	0.165	0.81 (0.60–1.09)
		AA+GA vs. GG			0.389	0.85 (0.58–1.24)			0.350	1.16 (0.85–1.58)	0.963	1.01 (0.80–1.27)
		AA vs. GG+GA			**0.022**	**0.59 (0.37–0.93)**			0.588	0.91 (0.64–1.29)	0.038	0.76 (0.58–0.99)

a P value was calculated by adjusted age and sex.

In the verification stage, as the *P* value was cut-off for 0.00128, we only found that the *XPC* rs1870134 GG genotype showed a significant increased risk for HCC (*P* = 4.7 × 10^−4^, OR = 1.67) when compared with CC + GC genotype. We merged this two stages for a meta-analysis, and also found this *XPC* rs1870134 GG genotype showed a significant increased risk for HCC (*P* = 0.001, OR = 1.45, Table 1).

And we also analyzed the association of the positive *XPC* rs1870134 SNP with the clinical features of HCC about smoking, drinking, family history, HBV and HCV infection status and histopathology classification, but found no significant association (Supplementary Table S3).

### The association of haplotype in NER pathway genes with hepatocellular cancer risk

We considered that haplotypes with a frequency less than 0.03 would be excluded from analysis. Six haplotypes in four genes were found to be associated with HCC risk.

Compared with other haplotypes, patients with the A-C-A-T haplotype of *DDB2* rs2029298-rs830083-rs3781619-rs326222 showed a 2.29-fold increased HCC risk (*P* = 0.007, 95% CI = 1.23–4.25), while patients with the G-C-C-C-T-C-G-G haplotype of *ERCC5* rs2094258-rs751402-rs2296147-rs1047768-rs4150291-rs2228959-rs4150383-rs873601 showed a significant protective function for HCC (*P* = 0.015, OR = 0.41, 95% CI = 0.20–0.86). As shown in Table 1, the promising SNPs associated with HCC risk were *ERCC1* rs2298881-rs3212961, and we only analyzed haplotypes composed of these positive SNPs instead of the analysis for *ERCC1* rs2298881-rs11615-rs3212955-rs3212961-rs3212986-rs735482, which simplified the analysis and obtained similar results to a complete analysis of all six SNPs. The A-C haplotype of *ERCC1* rs2298881-s3212961 had a protective function for HCC risk (OR = 0.70), while the C-C haplotype showed an increased risk (OR = 1.47; Table 2).

**Table 2:** The association of haplotype of each gene in NER pathways with hepatocellular cancer risk

Haplotype	Case (%)	Control (%)	*P*	OR (95% CI)
**Stage 1**				
**XPA^a^**				
C C T	75.83 (22.4)	199.47 (20.2)	0.376	1.15 (0.85–1.54)
T C C	168.83 (49.9)	490.28 (49.6)	0.906	1.02 (0.79–1.30)
T C T	63.17 (18.7)	201.56 (20.4)	0.502	0.90 (0.66–1.23)
T T T	29.00 (8.6)	94.14 (9.5)	0.607	0.89 (0.58–1.38)
**XPC^b^**				
C C C A	83.99 (24.8)	273.12 (27.9)	0.273	0.85 (0.64–1.13)
C G C C	125.90 (37.2)	350.22 (35.7)	0.630	1.07 (0.82–1.38)
C G T A	105.09 (31.1)	306.45 (31.3)	0.937	0.99 (0.76–1.29)
G G C A	18.00 (5.3)	34.07 (3.5)	0.134	1.56 (0.87–2.80)
**DDB2^c^**				
A C A T	18.45 (5.5)	24.29 (2.4)	**0.007**	**2.29 (1.23–4.25)**
A G G C	91.43 (27.2)	250.03 (25.2)	0.536	1.09 (0.83–1.45)
A G G T	11.47 (3.4)	34.04 (3.4)	0.959	0.98 (0.50–1.94)
G C A T	182.55 (54.3)	586.56 (59.1)	0.075	0.80 (0.62–1.02)
G G G C	15.57 (4.6)	27.83 (2.8)	0.112	1.66 (0.88–3.13)
G G G T	12.53 (3.7)	46.03 (4.6)	0.459	0.79 (0.42–1.49)
**ERCC3^d^**				
A A C	45.00 (13.3)	97.98 (10.0)	0.102	1.37 (0.94–2.00)
A G T	94.00 (27.8)	307.00 (31.2)	0.189	0.83 (0.63–1.10)
G G C	197.00 (58.3)	559.98 (56.9)	0.848	1.03 (0.80–1.32)
**ERCC2^e^**				
G G C G C T	15.63 (4.6)	98.40 (10.1)	**0.001**	**0.42 (0.24–0.72)**
G G T G C T	7.98 (2.4)	29.70 (3.1)	0.469	0.75 (0.34–1.65)
G T C G C T	70.07 (20.7)	243.34 (25.1)	0.065	0.75 (0.55–1.02)
G T C G T G	17.12 (5.1)	21.31 (2.2)	**0.009**	**2.33 (1.22–4.46)**
G T T G C T	31.77 (9.4)	66.46 (6.9)	0.154	1.38 (0.89–2.15)
T G C C C T	50.08 (14.8)	125.89 (13.0)	0.475	1.14 (0.80–1.63)
T T C C C T	83.02 (24.6)	186.46 (19.2)	0.054	1.34 (0.99–1.81)
T T C G C T	8.16 (2.4)	33.87 (3.5)	0.303	0.67 (0.31–1.45)
T T T C C T	20.03 (5.9)	46.96 (4.8)	0.487	1.21 (0.71–2.08)
**ERCC1^g^**				
A C A A G C	117.91 (35.1)	379.68 (38.4)	0.256	0.86 (0.66–1.12)
A C G C T A	22.99 (6.8)	107.79 (10.9)	**0.030**	**0.60 (0.37–0.95)**
A T A C G A	23.94 (7.1)	79.90 (8.1)	0.569	0.87 (0.54–1.40)
C C G C T A	71.68 (21.3)	176.21 (17.8)	0.150	1.26 (0.92–1.72)
C T A C G A	63.00 (18.8)	137.02 (13.9)	0.029	1.45 (1.04–2.02)
**ERCC1^h^,^k^**				
A-A	126.00 (37.3)	397.00 (39.9)	0.401	0.90 (0.70–1.16)
A-C	54.00 (16.0)	213.00 (21.4)	**0.032**	**0.70 (0.50–0.97)**
C-A	18.00 (5.3)	62.00 (6.2)	0.547	0.85 (0.49–1.45)
C-C	140.00 (41.4)	324.00 (32.5)	0.003	1.47 (1.14–1.89)
**ERCC4^i^**				
C T G	76.96 (22.9)	213.55 (21.9)	0.722	1.06 (0.79–1.42)
T C C	69.96 (20.8)	201.96 (20.7)	0.986	1.00 (0.74–1.36)
T T G	188.04 (56.0)	553.45 (56.8)	0.754	0.96 (0.75–1.23)
**ERCC5^j^**				
A C T T A C G G	128.41 (38.2)	343.38 (37.2)	0.967	1.01 (0.77–1.31)
G C C C A C G A	39.12 (11.6)	99.23 (10.8)	0.751	1.07 (0.72–1.58)
G C C C T C G A	8.48 (2.5)	53.16 (5.8)	**0.015**	**0.41 (0.20–0.86)**
G C C C T C G G	10.20 (3.0)	31.49 (3.4)	0.691	0.87 (0.42–1.77)
G C T C A C G G	25.26 (7.5)	58.60 (6.4)	0.525	1.17 (0.72–1.90)
G T T T A A G A	10.65 (3.2)	33.68 (3.7)	0.633	0.84 (0.42–1.70)
G T T T A C A A	22.72 (6.8)	60.94 (6.6)	0.999	1.00 (0.61–1.65)
G T T T A C G A	54.18 (16.1)	144.30 (15.7)	0.960	1.01 (0.72–1.42)
G T T T A C G G	21.07 (6.3)	34.06 (3.7)	0.058	1.71 (0.98–2.99)
**Stage 2**				
**ERCC1^k^**				
A–A	387 (39.0)	657.78 (37.5)	0.430	1.07 (0.91–1.25)
C–A	63.1 (6.3)	140.22 (8.0)	0.113	0.78 (0.57–1.06)
C–C	522.90 (52.6)	940.78 (53.6)	0.603	0.96 (0.82–1.12)

Haplotype for^a^, XPA rs10817938-rs3176629-rs2808668;^b^, XPC rs2607775-rs1870134-rs2228000-rs2228001;^c^, DDB2 rs2029298-rs830083-rs3781619-rs326222;^d^, ERCC3 rs4150441-rs4150448-rs4150506;^e^, ERCC2 rs238406-rs50871-rs50872-rs238417-rs1052555-rs13181;g, ERCC1 rs2298881-rs11615-rs3212955-rs3212961-rs3212986-rs735482;h, ERCC1 rs2298881-rs3212961;^i^, ERCC4 rs254942-rs1799801-rs2276464;^j^, ERCC5 rs2094258-rs751402-rs2296147-rs1047768-rs4150291-rs2228959-rs4150383-rs873601.k, ERCC1 rs2298881-rs3212961 according to the main analysis in Table 1, we further simplified the analysis for ERCC1 and ERCC2 haplotype and only analyzed the haplotype of the positive ERCC1 rs2298881-rs3212961, which got a similar result with the full one.

Linkage disequilibrium data composed of D' and *r*^2^ for these eight polymorphisms are shown in Supplementary Figure S1.

### Gene-gene interaction models for polymorphisms in NER pathways

In order to analyze the best interaction model composed of the six promising SNPs in NER pathways genes in stage 2, we used MDR software to select the best model for gene-gene interaction (Table 3). To predict HCC risk, the best interaction model selected from the six positive SNPs was the four-factor model including *ERCC1* rs2298881 *-XPC* rs1870134-*ERCC2* rs238417-*ERCC5* rs873601 SNPs, which yielded the highest testing accuracy of 0.6001 and the maximal CV consistency of 10/10 (significant test *P* = 0.0010, and *P* for permutation test = 0.0010–0.0020). And the second interaction model selected was the two-factor model including *ERCC1* rs2298881-*XPC* rs1870134, which yielded the highest testing accuracy of 0.5977 and the maximal CV consistency of 10/10 (significant test *P* = 0.0107, and *P* for permutation test = 0.0010–0.0020).

**Table 3:** Gene-gene interaction models for polymorphisms in NER pathways for hepatocellar cancer risk by MDR analysis

Model	Training Bal. Acc.	Testing Bal. Acc.	Sign Test (*p*)	CV Consistency	*P* for permutation test
XPC rs18701343	0.5798	0.5803	8 (0.0547)	10/10	0.0190
ERCC1 rs2298881-XPC rs1870134^b^	0.5974	0.5977	9 (0.0107)	10/10	**0.0010–0.0020**
ERCC1 rs2298881-ERCC1 rs3212961-XPC rs1870134	0.6061	0.5689	9 (0.0107)	4/10	0.0640
ERCC1 rs2298881-XPC rs1870134-ERCC2 rs238417-ERCC5 rs873601^a^	0.6218	0.6001	10 (0.0010)	10/10	**0.0010–0.0020**

Note: The best model was selected as the one with the maximum testing accuracy and maximum CV Consistency.

a In this study, the best interaction model was the four-factor model including ERCC1 rs2298881-XPC rs1870134-ERCC2 rs238417-ERCC5 rs873601 polymorphisms.

b , the second best interaction model was the two-factor model including ERCC1 rs2298881-XPC rs1870134 polymorphisms.

To further validate the MDR results, we conducted analyses of both four-factor and two-factor models using the multivariate logistic regression, and found *ERCC1* rs2298881-*XPC* rs1870134 pairwise interaction to be significant (*P* = 0.023, OR = 2.11, 95% CI = 1.11–4.03; Table 4). However, the four-factor model interaction was not reached to statistical significance (*P*_interaction_ = 0.340).

**Table 4:** The genotype combinations of the SNP-SNP interactions in two polymorphisms with the risk of hepatocellular cancer^a^

SNP genotypes		CON	HCC	*P*	OR (95% CI)
ERCC1 rs2298881	XPC rs1870134				
CC	CC+GC	60	44		1 (Ref.)
CC	GG	80	57	0.913	0.97 (0.58–1.63)
CA + AA	CC+GC	119	73	0.472	0.84 (0.52–1.36)
CA + AA	GG	111	142	0.018	1.74 (1.10–2.77)
		***P*_interaction_** = **0.023, 95% CI = 2.11 (1.11–4.03)**					

Note:

a , *P* for interaction was used Logistic Regession adjusted by sex and age. CON: controls; HCC: hepatocellular cancer.

### Differences of XPC rs1870134 gene mRNA levels in different genotypes in hepatocellular cancer and non-cancer tissues

For *XPC* mRNA expression level in non-cancerous and cancerous tissues, we found *XPC* gene was decreased in tend from non-cancer tissues to cancer tissues (1.50 ± 4.32 vs. 3.58 ± 25.77, Table 5), although the *P* value did not reach the statistical significance (*P* = 0.456). We further explore the potential biological significance of the *XPC* rs1870134 polymorphism at mRNA level (Table 5). In cancerous group, *XPC* mRNA levels were significantly lower in subjects carrying *XPC* rs1870134 GG genotype compared with patients with CC genotype (*P* = 5 × 10^−5^).

**Table 5:** Differences of XPC gene mRNA levels in different genotypes in hepatocellular cancer and non-cancer tissues

Variable	Non-cancer tissue				Cancer tissue			
	*N*(86)	ΔCt(Mean ± SD)	Normalized 2−ΔΔCt	*P*	*N*(86)	ΔCt (Mean ± SD)	Normalized 2-ΔΔCt	*P*
For XPC mRNA		1.50 ± 4.32	1 (0.05,19.97)			3.58 ± 25.77	0.24 (0.01,4.72)	0.456
The effect of XPC rs1870134 genotypes to XPC mRNA								
CC	3	0.24 ± 0.12	1 (0.92,1.09)	Ref.	3	0.25 ± 0.05	1 (0.97,1.04)	Ref.
GC	40	4.65 ± 1.74	0.05 (0.01,0.16)	0.587	40	6.77 ± 38.20	0.01 (0.00,3 × 10^9^)	0.772
GG	43	0.91 ± 0.75	0.63 (0.37,1.06)	**5 × 10^−5^**	43	0.81 ± 1.25	0.67 (0.28,1.60)	0.451

## DISCUSSION

The NER repair pathway was divided into three steps: the recognition-unwinding-incision steps. Briefly, in the recognition step, XPC-RAD23B complex or UV-damaged DNA-binding protein 2 (DDB2) could recognize DNA damage. In the unwinding step, Helicase subunits composed of XPB (ERCC3) and XPD (ERCC2) were activated, and opened the DNA duplex, then recruited XPA. XPB was a 3′ to 5′ translocase and XPD was a 5′ to 3′ translocase. In the incision step, XPD (ERCC2) remained in the damaged DNA 3′ region, and then XPG (ERCC5) cut on the 3′ side while ERCC1-XPF (ERCC4) complex on the 5′ side. The damaged DNA was then repaired. Although several studies have reported an association between a single exon SNP in NER gene (such as *XRCC1* Arg399Gln) with HCC, none have demonstrated a systematic and comprehensive analysis between polymorphisms in every step of NER pathways genes and HCC risk. We preliminarily screened among the NER pathways SNPs for HCC risk, and identified three risk SNPs and three protective SNPs in five genes, that is, the positive six SNPs composed of two in recognition step (*XPA* rs10817938 and *XPC* rs1870134), one in unwinding step (*ERCC2* rs238417) and three in incision step (*ERCC5* rs873601, *ERCC1* rs2298881 and rs3212961), and one combination of a gene–gene interaction model (*ERCC1* rs2298881-*XPC* rs1870134 pairwise) associated with HCC risk. Further functional experiments confirmed that one positive SNP *XPC* rs1870134 associated with HCC risk had an effect of polymorphism on *XPC* mRNA expression.

### XPA rs10817938 and XPC rs1870134 in recognition step

XPA was the first human NER protein showing a preference for binding to damaged DNA. It is also a zinc-binding protein with affinity for various DNA damage [14] that functions at the core of the NER system [15]. *XPA* is located on chromosome 9q22.3, and the promoter rs10817938 SNP we studied is located in −2718 bp from the transcription start site. This polymorphism has not been evaluated to be associated with the risk of any disease, but another *XPA* promoter SNP located at −4 bp might change *XPA* mRNA tertiary structure and stability, and might play a role in susceptibility to cancer [16]. The association between *XPA* polymorphisms with HCC risk is biologically plausible since XPA plays an important role in NER pathway while XPA protein defects were previously shown to lead to HCC susceptibility in mouse model experiments [6]. And the XPC protein also plays an important role early in the DNA damage recognition step. It tightly binds to a distorted region and changes the structure of the DNA to allow other components of the repair apparatus to enter [15]. *XPC* gene is located on chromosome 3q25, and the promoter rs1870134 SNP we studied is located at +149 bp, which has not previously been reported to be associated with disease. In this study, we found two promoter SNPs in the *XPA* and *XPC* genes respectively were shown to be associated with HCC risk. As mentioned above, the promoter SNP could change the gene's function, including mRNA structure or stability, and protein expression or function, which would explain our observed association of this two promoter SNPs with HCC susceptibility.

Because the *XPC* gene promoter rs1870134 SNP was confirmed by the verification stage, we further performed *XPC* mRNA expression study. We found the expression of *XPC* gene was decreased in cancer tissues when compared with non-cancer tissues, which suggest that XPC protein functioned as protective protein in hepatocellular cancer. Then, we analyzed the effects of this SNP on *XPC* mRNA expression in order to clarify the possible mechanism for polymorphisms. We found the GG genotype which was associated with an increased HCC risk, showed a lower *XPC* mRNA expression. As XPC was a protective protein, individuals carrying risk GG genotype showed a decreased mRNA expression, causing the lower expression of this protective protein, which might be the possible mechanism for the high risk of GG genotype. The similar study reported that the rs2298881 G allele of *ERCC1* gene located in 5′-flanking region was associated with a down-regulated protein expression, and subsequent functional experiment showed this SNP could decrease the gene's promoter activity and transcription factor binding activity [17]. Thus, further functional experiments such as promoter activity and transcription factor binding activity assays should be performed to clarify the associated mechanism.

### ERCC2 rs238417 in unwinding step

ERCC2, also called XPD, is an unwinding protein in NER repair that unwinds DNA from 5′ to 3′ [15]. *ERCC2* gene is located on chromosome 19q13.3, and includes 21 SNP sites in the HapMap database. *ERCC2* SNP rs238417 is located in intron 18 which was short and only 90 bp length. Thus any polymorphism among intron 18 could largely change the second structure and mRNA stability, and the variation of rs238417 from G to C could make 3 kcal/mol change of the minimum free energy from stable to unstable using RNAfold predicting software (http://rna.tbi.univie.ac.at/cgi-bin/RNAfold.cgi). Peethambaram *P* et al. studied 11 SNPs in *ERCC2* gene and found this rs238417 SNP was the most significant polymorphism associated with the outcome of ovarian cancer [18]. In the present study, we demonstrated that the rs238417 variant CC genotype increased the HCC risk by 1.77-fold. As previous study showed, an intronic polymorphism also played an important role in mRNA splicing or protein expression [19]. Considering the length of this intron 18 was short, the short intron may cause this polymorphism to be a functional SNP. The above-mentioned might be the reason for our observed association of this rs238417 SNP with HCC susceptibility. Further experiments are required to confirm this observation.

### ERCC1 rs2298881 and rs3212961 in incision step

ERCC1 is an incision and repair protein that binds first to ERCC4 (XPF), then ERCC3 (XPB). This ERCC1–ERCC4–ERCC3 complex cuts DNA from 5′ to 3′ [15]. *ERCC1* gene is located on chromosome 19q13.32 and any variation of *ERCC1* might cause the change of ERCC1 protein, ERCC1–ERCC4–ERCC3 complex and even the whole NER pathway. Yin J et al. studied several SNPs in *ERCC1* and *ERCC2* and found both the rs2298881 and rs3212961 SNPs had interaction with smoking in lung cancer patients [20]. We also found that *ERCC1* rs2298881 and rs3212961 were significantly associated with HCC risk. Several studies have previously reported an association with the *ERCC1* promoter SNP rs2298881 and cancer risk. Indeed, Yu et al. found this rs2298881 SNP was associated with a decreased risk of lung cancer [17]. Other study showed that this SNP exhibited an increased prostate cancer risk with high fonofos exposure compared with controls [21]. In this study, we found that the *ERCC1* promoter rs2298881 CA heterozygote decreased the HCC risk by a 0.64-fold, which was consistent with the report by Yu et al. It was reported that the variant allele of this rs2298881 down-regulated *ERCC1* promoter activity, down-regulated transcription factor binding activity, and thus decreased ERCC1 protein expression with a subsequent decrease in the cancer risk [17]. Another HCC-associated *ERCC1* SNP, rs3212961, located in intron 3 immediately 3′ to exon 3, could affect transcription splicing, because *ERCC1* shows several splicing variants. Although many studies showed the association of this SNP with cancer risk, the results were inconsistent. Shen et al. found that rs3212961 decreased the risk of lung cancer under a dominant model [22], while others showed that it could increase the risk for bladder cancer under a dominant model [23], as well as colorectal cancer [24]. In this study, we demonstrated that rs3212961 decreased HCC risk by 0.66-fold under the dominant model, but the detailed underlying mechanism should be investigated, especially whether this polymorphism influence the expression of *ERCC1* exon 3 or influence the selective splicing.

### ERCC5 rs873601 in incision step

ERCC5 (XPG) is activated and binds the 3′ region of DNA damage. This is followed by ERCC2 and ERCC5 (XPG) binding. *ERCC5* rs873601 SNP is located in the 3′-UTR. Regarding *ERCC5* rs873601 SNP, a previous report showed no association with the risk of esophageal squamous cell carcinoma [25]. In single locus analysis, we found this SNP could decrease HCC risk. We speculate the way of the possibilities: 1) impact through ERCC5 mRNA and protein, or 2) impact through a third party. It is possible that this polymorphism affect protein expression and/or activity, or that they involve the same miRNA that binds the 3′ UTR region. Based on this result, we recently performed the mRNA expression experiment to further analyze the effects of SNP genotypes to the mRNA expression. The *ERCC5* rs873601 variant genotypes associated with a decreased HCC risk showed a higher mRNA expression of *ERCC5* gene (Data were not published). The mechanism of the rs873601 SNP to the mRNA level expression was not very clear now, so further functional study is required to verify this finding in future studies.

### Gene haplotype with HCC risk

Combined haplotype and LD association analyses for multiple SNPs are more sensitive and powerful than SNP analysis alone [26]. We showed that the *DDB2* A-C-A-T haplotype of rs2029298-rs830083-rs3781619-rs326222 increased the HCC risk (OR = 2.29), while these SNPs alone had no significant association with HCC risk, indicating that the haplotype was more sensitive than a single SNP. We re-analyzed a positive SNP detected in single-site analysis of *ERCC1*, and observed that the two-site combination haplotype showed similar results to the entire six-site combination haplotype for both *ERCC1*. This simplification showed that the *ERCC1* C-C haplotype of rs2298881-rs3212961 increased HCC risk, while the *ERCC1* A-C haplotype decreased HCC risk.

### SNP-SNP interaction with HCC risk

One of the most significant finding in our study was the multiple SNP–SNP interactions composed of *ERCC1* rs2298881 and *XPC* rs1870134 polymorphisms, which were consistently identified by two different statistical approaches: multivariate logistic regression and MDR analyses. We found the *P* value for “*ERCC1* rs2298881-*XPC* rs1870134-*ERCC2* rs238417-*ERCC5* rs873601” combination was more significant than the two-way interactions of “*ERCC1* rs2298881 and *XPC* rs1870134”, but the four-way interaction combination was not verified by the multivariate logistic regression method, which might due to the more subgroups causing the rare genotypes. Several studies showed that the combined effect of multiple SNPs in several genes in one or more relevant DNA repair pathways could have a greater impact on pathological phenotypes than SNPs in single genes [27]. And we found the OR of “*ERCC1* rs2298881 and *XPC* rs1870134” polymorphisms interaction was higher than the OR of single-locus (OR _interaction_: 2.11 vs. OR *XPC*: 1.67), which suggest that this two-way interaction was a superior combination model for the prediction of HCC risk. As the mechanism of these two SNPs was not very clear now, it required further functional study to verify this finding in future studies.

### Limitations

However, this study still had some limitations. First, the sample size was still limited, thus restricted the probability of the subgroup analysis for variant genotypes, and also limited the interaction analysis. Second, the HCC samples were only from the surgically resected patients and not covered for patients undergoing radiotherapy or chemotherapy. However, maybe because of this, we avoided and eliminated the effect of the heterogeneity bringing from the samples sourced from various treatments. Third, the expression of the promising *XPC* gene in this study was only at mRNA level, while the protein expressions were also warranted to study in future research.

### Conclusion

In summary, we preliminarily explored gene polymorphisms in NER pathway for predicting the risk of HCC. In the screening stage, six SNPs of five genes in three steps of NER pathways were associated with HCC risk, including three risk SNPs (*XPA* rs10817938, *XPC* rs1870134 and *ERCC2* rs238417) and three protective SNPs (*ERCC1* rs2298881 and rs3212961, and *ERCC5* rs873601). In the verification stage, only *XPC* rs1870134 was verified to be associated with an increased HCC risk. Furthermore, multivariate logistic regression and MDR analyses consistently revealed the SNP–SNP interaction comprised of *ERCC1* rs2298881 and *XPC* rs1870134 pairwise was associated with HCC risk. Further analysis for the effect of polymorphism on mRNA expression suggested that *XPC* rs1870134 GG genotype which associated with an increased HCC risk demonstrated a lower *XPC* mRNA expression. Future large-scale samples and functional molecular experiments are required to confirm our results.

## MATERIALS AND METHODS

### Patients

This research project was approved by the Ethical Committee of the First Affiliated Hospital of the China Medical University and written informed consent was obtained. The study was designed based on two-stage for the aim of “screening-verification”. All clinical investigations have been conducted according to the principles expressed in the Declaration of Helsinki. This study comprised of a total of 1472 participants, among them, 570 patients (included 169 and 401 HCC patients for the screening stage and verification stage, respectively) were underwent surgical operation for HCC at the First Affiliated Hospital of China Medical University between 2012 and 2015. The participants underwent surgical operation were diagnosed by pathological confirmation with HCC according to WHO classification. A total of 902 controls were recruited from a health screening program from the Zhuanghe area, Liaoning Province, China between 2002–2012. Among them, for the screening stage, 501 controls were 1:3 frequency matched with the 169 cases of HCC according to the condition of the matched sex (1:1) and age (± 5). And for the verification stage, 401 controls were 1:1 matched with the 401 cases of HCC by the same condition. Peripheral venous blood specimen was collected from participants and stored at −20°C until use.

### Polymorphisms sites selected

We selected polymorphisms using HapMap data (http://www.broadinstitute.org/mpg/haploview) referred to previously [13, 28, 29], in order to minimize the number of SNPs required to be genotyped, providing an important shortcut to carry out candidate gene association studies in a particular population. The tag SNPs were selected separately using the following criteria: 1). using Haploview by Tagger function; 2). the population of the HapMap selected CHB (Chinese Han Beijing) population; 3). the pairwise tagging with *r*^2^ at least 0.8; 4). a minor allele frequency was at least 5%. The selection area was enlarged by 10 kb both upstream and downstream for all genes. FastSNP Search was used to predict the potential SNP function (leading to amino acid substitutions, altering splicing or transcription factor-binding motifs, acting as intronic enhancers) [30, 31]. A total of 39 SNPs covering eight key NER pathway genes were selected by integrating these two publicly available tools. Locations and characterizations of the selected SNPs are shown in Table 1.

### Genotyping

Genomic DNA was extracted using a previously published method [32] and diluted to working concentrations of 20 ng μL^−1^ for genotyping. The genotyping assay for the first stage was performed by Bomiao Biological Company (Beijing, China) using the Sequenom MassARRAY platform (Sequenom, San Diego, CA, USA). In the second stage (verification stage), the assay was performed by Gene Company (Shanghai, China), using allele-specific PCR using KASPar (KASP) reagents (LGC Genomics, Hoddesdon, UK). For quality control, we repeatedly genotyped 10% of the total samples at one time in each stage, and beside these, we verified genotyping results in a third method by Huada Gene Company using direct sequencing for another 10% random samples. The concordance rate of these repeated samples reached 100%, which demonstrated that the genotyping results were reliable.

### Quantitative RT-PCR analysis for the mRNA expression of promising genes

The TRIzol reagent was used to isolate total RNA from approximately 50 mg tissues in 86 HCC specimens and their neighboring 86 non-cancer samples (Life Technologies, Carlsbad, CA, USA). The method of this part was described previously [28], and a total of 2.0 μg isolated RNA was converted into cDNA using Quantscript RT Kit (Tiangen Biotech, Beijing, China). The mRNA expression of the aimed promising genes and an internal-control gene GAPDH were tested using SYBR Premix Ex Taq II (TaKaRa Biotech, Dalian, China) in an Eppendorf Mastercycler Gradient System (Eppendorf AG, Hamburg, Germany). Each reaction was performed in duplicates and blank controls without cDNA template were also tested every time. The primers were summarized in Supplementary Table S4.

The relative quantification of the mRNA level was calculated using 2^−ΔΔCt^ method [33]. The expression levels of the aimed promising genes were normalized to those of GAPDH in each sample using the equation: ΔCt (delta Ct)=Ct_target_-Ct_GAPDH_. Relative expression levels were derived from ΔCt-values as 2^−ΔCt^. For patients stratified by the genotypes, the relative expression of patients with the common homogenous genotype were set to a unity, and the relative expression of heterogeneous and rare homogenous genotypes carriers were expressed relative to those of patients with the common homogenous *g* enotype, thus deriving normalized 2^−ΔΔCt^ values.

### Statistical analysis

Between-group differences in sex variability, as well as the Hardy Weinberg Equilibrium, were compared by the χ^2^ test. And analysis of variance was used for age variability. Multivariate logistic regression with adjustments for age and sex was used to show the association between selected gene polymorphisms with HCC risk. The haplotype of each gene was analyzed using SHEsis software [34]. All NER gene polymorphisms identified in the best models of gene–gene interactions were calculated using MDR software (version 3.0.2) and MDR permutation testing software (version 1.0 beta 2) [35]. The combined effect of selected SNP–SNP interactions in the best model was determined by multivariate logistic regression adjusted for age and sex. The differences of relative mRNA levels between two groups were tested by the Student *t*-test. In the screening stage, *P* value < 0.05 was considered significant. And significance values shown for the analysis in stage 2 and merged meta data were adjusted for multiple test correction. The cut-off of significance *P* value was used as < 0.00128 (0.05 ÷ 39 = 0.00128) for the verification stage.

## SUPPLEMENTARY MATERIALS FIGURE AND TABLES



## Abbreviations

SNP: single nucleotide polymorphism
OR: odds ratio
CI: confidence interval
HCC: hepatocellular cancer
NER: Nucleotide Excision Repair.
