# Supplementary material for: The association of six polymorphisms of five genes involved in three steps of nucleotide excision repair pathways with hepatocellular cancer risk
Source: Oncotarget. 2016 Mar 7;7(15):20357–67. doi: 10.18632/oncotarget.7952 (PMC4991460; doi:10.18632/oncotarget.7952)
Supplement: Supplementary file 1 [file oncotarget-07-20357-s001.pdf]

The association of six polymorphisms of five genes involved in three steps of nucleotide excision repair pathways with hepatocellular cancer risk

Supplementary Materials

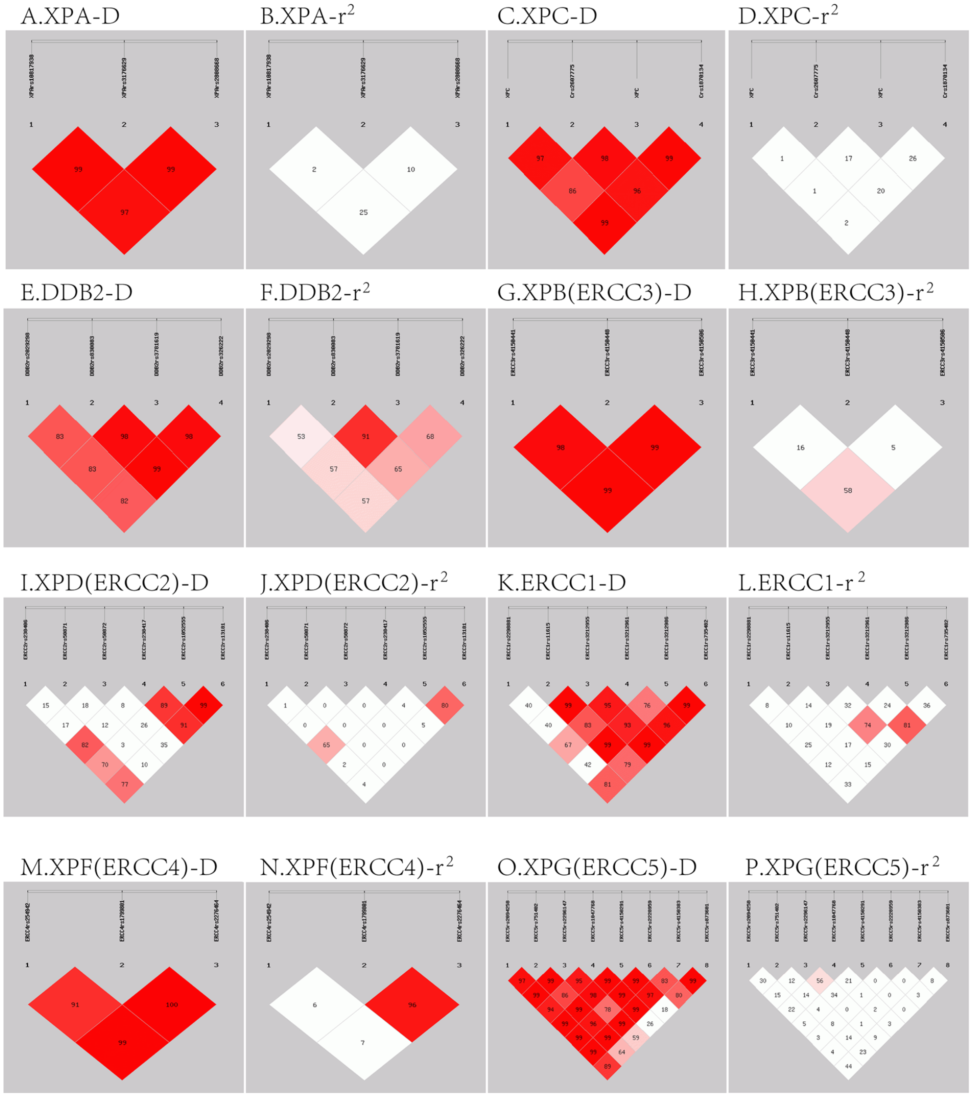

Supplementary Figure S1: The linkage disequilibrium data including D' and r^2 of these 39 polymorphisms in 8 genes. The figures in each cell tables meant the value of D' or r^2, and degree of linkage disequilibrium showed as the deepness of the color (form the red to the white).

Supplenmentary Table S1: The baseline of the subjects

Variables	Hepatocellular cancer patients (%)	Controls (%)	*P*	Hepatocellular cancer patients (%)	Controls (%)	*P*
	n = 169	*n* = 501		*n* = 401	*n* = 401	
Gender			0.950			0.070
Male	139 (82.2)	411 (82.0)		326 (81.3)	305 (76.1)	
Female	30 (17.8)	90 (18.0)		75 (18.7)	96 (23.9)	
Age			0.603			0.853
Mean ± SD	57.5 ± 9.8	57.1 ± 9.4		56.6 ± 11.2	56.7 ± 10.9	
Median	57	57		58	57	
Range	34–86	34–86		21–90	17–89	
Smoking			**< 0.001**			0.086
Never	123 (78.3)	168 (52.2)		265 (71.4)	93 (63.7)	
Ever	34 (21.7)	154 (47.8)		106 (28.6)	53 (36.3)	
Drinking			**< 0.001**			0.119
Never	129 (82.2)	204 (63.6)		309 (83.3)	113 (77.4)	
Ever	28 (17.8)	117 (36.4)		62 (16.7)	33 (22.6)	
Cancer Family History						
Had	24 (14.2)	ND		75 (18.7)	ND	
No	135 (79.9)	ND		296 (73.8)	ND	
Missing Data	10 (5.9)	ND		30 (7.4)	ND	
HBsAg						
Negative	36 (22.0)	ND		269 (78.0)	ND	
Positive	128 (78.0)	ND		76 (22.0)	ND	
Anti–HCV						
Negative	149 (91.4)	ND		135 (76.3)	ND	
Positive	14 (8.6)	ND		42 (23.7)	ND	

ND, not known.

Supplementary Table S2: The association of NER pathway gene SNPs and risk of hepatocellular cancer

Gene	Chr. Pos.	SNP*	Loc.	Genotype	Cases (%)	Controls (%)	*P*†	OR (95% CI)
**Recognition–related**								
XPA	9q22.3	rs10817938	Promoter	TT	105 (62.1)	311 (62.1)		
				TC	51 (30.2)	172 (34.3)	0.525	0.88 (0.60–1.30)
				CC	13 (7.7)	15 (3.0)	**0.020**	**2.52 (1.16–5.49)**
		rs3176629	Promoter	CC	142 (84.0)	407 (81.2)		
				CT	25 (14.8)	86 (17.2)	0.478	0.84 (0.52–1.36)
				TT	2 (1.2)	6 (1.2)	0.949	0.95 (0.19–4.77)
		rs3176658	Intron 2	CC	169 (100.0)	497 (99.2)		
		rs2808668	Intron 2	CC	43 (25.4)	114 (22.8)		
				CT	84 (49.7)	265 (52.9)	0.434	0.84 (0.55–1.29)
				TT	42 (24.9)	116 (23.2)	0.895	0.97 (0.59–1.60)
XPC	3p25	rs2607775	Promoter	CC	151 (89.3)	465 (92.8)		
				CG	17 (10.1)	35 (7.0)	0.189	1.51 (0.82–2.77)
				GG	1 (0.6)	1 (0.2)	0.443	2.98 (0.18–48.42)
		rs1870134	Promoter	GG	88 (52.1)	257 (51.3)		
				GC	76 (45.0)	204 (40.7)	0.654	1.09 (0.76–1.56)
				CC	5 (3.0)	39 (7.8)	**0.048**	**0.39 (0.14–0.99)**
		rs2228000	Exon 8	CC	79 (46.7)	225 (44.9)		
				CT	74 (43.8)	222 (44.3)	0.813	0.96 (0.66–1.38)
				TT	16 (9.5)	46 (9.2)	0.977	0.99 (0.53–1.85)
		rs2228001	Exon 15	AA	68 (40.2)	200 (39.9)		
				AC	74 (43.8)	234 (46.7)	0.733	0.94 (0.64–1.37)
				CC	27 (16.0)	63 (12.6)	0.415	1.25 (0.73–2.12)
		rs2470352	3′–UTR	AA	169 (100.0)	497 (99.2)		
				AT	0 (0.0)	3 (0.6)	NA	NA
				TT	0 (0.0)	0 (0.0)	0.000	0
DDB2	11p12	rs2029298	Promoter	GG	64 (37.9)	233 (46.5)		
				GA	86 (50.9)	218 (43.5)	0.060	1.43 (0.99–2.08)
				AA	19 (11.2)	50 (10.0)	0.290	1.38 (0.76–2.51)
		rs830083	Intron 3	CC	60 (35.5)	188 (37.5)		
				CG	82 (48.5)	243 (48.5)	0.775	1.06 (0.72–1.55)
				GG	27 (16.0)	69 (13.8)	0.452	1.23 (0.72–2.09)
		rs3781619	Intron 4	AA	60 (35.5)	198 (39.5)		
				AG	86 (50.9)	238 (47.5)	0.359	1.20 (0.82–1.75)
				GG	23 (13.6)	63 (12.6)	0.512	1.21 (0.69–2.11)
		rs326222	3′ near region	TT	78 (46.2)	245 (48.9)		
				TC	73 (43.2)	224 (44.7)	0.932	1.02 (0.70–1.47)
				CC	17 (10.1)	30 (6.0)	0.084	1.77 (0.93–3.39)
**Unwinding–related**								
XPB(ERCC3)	2q21	rs4150441	Intron 8	GG	59 (34.9)	165 (32.9)		
				GA	80 (47.3)	235 (46.9)	0.789	0.95 (0.64–1.40)
				AA	30 (17.8)	97 (19.4)	0.564	0.86 (0.52–1.43)
		rs4150448	Intron 8	GG	127 (75.1)	405 (80.8)		
				GA	39 (23.1)	86 (17.2)	0.089	1.45 (0.95–2..23)
				AA	3 (1.8)	8 (1.6)	0.832	1.16 (0.30–4.45)
		rs4150506	Intron 12	CC	90 (53.3)	232 (46.3)		
				CT	63 (37.3)	217 (43.3)	0.123	0.75 (0.52–1.08)
				TT	16 (9.5)	48 (9.6)	0.617	0.85 (0.46–1.58)
XPD(ERCC2)	19q13.3	rs238406	Exon 5	GG	42 (24.9)	147 (29.3)		

				GT	85 (50.3)	253 (50.5)	0.482	1.16 (0.76–1.78)
				TT	42 (24.9)	98 (19.6)	0.152	1.45 (0.87–2.39)
		rs50871		TT	95 (56.2)	204 (40.7)		
			Intron 12 in transcript 1; 3′ near region in transcript 2	TG	62 (36.7)	269 (53.7)	**1.87×10^{-4}**	**0.50 (0.34–0.72)**
				GG	12 (7.1)	28 (5.6)	0.777	0.90 (0.44–1.86)
		rs50872		CC	110 (65.1)	316 (63.1)		
			Intron 12 in transcript 1; 3′ near region in transcript 2	CT	50 (29.6)	169 (33.7)	0.428	0.86 (0.58–1.26)
				TT	9 (5.3)	15 (3.0)	0.178	1.81 (0.76–4.29)
		rs238417	Intron 18	GG	39 (23.1)	149 (29.7)		
				GC	87 (51.5)	248 (49.5)	0.191	1.33 (0.87–2.05)
				CC	43 (25.4)	90 (18.0)	**0.028**	**1.77 (1.06–2.95)**
		rs1052555	Exon 21	CC	146 (86.4)	445 (88.8)		
				CT	23 (13.6)	54 (10.8)	0.326	1.30 (0.77–2.19)
				TT	0 (0.0)	2 (0.4)	NA	NA
		rs13181	3′–UTR	TT	138 (81.7)	436 (87.0)		
				TG	30 (17.8)	63 (12.6)	0.091	1.51 (0.94–2.42)
				GG	1 (0.6)	2 (0.4)	0.725	1.54 (0.14–17.20)
**Incision–related**								
ERCC1	19q13.32	rs2298881	Promoter	CC	79 (46.7)	194 (38.7)		
				CA	58 (34.3)	224 (44.7)	**0.027**	**0.64 (0.44–0.95)**
				AA	32 (18.9)	81 (16.2)	0.929	0.98 (0.60–1.59)
		rs11615	Exon 3	CC	87 (51.5)	288 (57.5)		
				CT	66 (39.1)	182 (36.3)	0.356	1.19 (0.82–1.73)
				TT	15 (8.9)	31 (6.2)	0.173	1.58 (0.82–3.07)
		rs3212955	Intron 3	AA	89 (52.7)	243 (48.5)		
				AG	60 (35.5)	211 (42.1)	0.217	0.79 (0.54–1.15)
				GG	20 (11.8)	47 (9.4)	0.583	1.18 (0.66–2.10)
		rs3212961		CC	64 (37.9)	143 (28.5)		
			Intron 3 immediately 3′ to the exon 3	CA	66 (39.1)	251 (50.1)	**0.012**	**0.60 (0.40–0.89)**
				AA	39 (23.1)	106 (21.2)	0.422	0.83 (0.51–1.32)
		rs3212986	3′–UTR	GG	78 (46.2)	226 (45.1)		
				GT	69 (40.8)	211 (42.1)	0.818	0.96 (0.66–1.39)
				TT	22 (13.0)	62 (12.4)	0.923	1.03 (0.59–1.78)
		rs735482	3′–UTR	CC	35 (20.7)	96 (19.2)		
				CA	64 (37.9)	235 (46.9)	0.227	0.75 (0.46–1.20)
				AA	70 (41.4)	168 (33.5)	0.589	1.14 (0.70–1.84)
XPF(ERCC4)	16p13.12	rs254942	Intron 5	TT	100 (59.2)	294 (58.7)		
				TC	58 (34.3)	173 (34.5)	0.928	0.98 (0.68–1.43)
				CC	10 (5.9)	22 (4.4)	0.471	1.34 (0.61–2.93)
		rs1799801	3′–UTR	TT	100 (59.2)	309 (61.7)		
				TC	65 (38.5)	166 (33.1)	0.328	1.20 (0.83–1.73)
				CC	4 (2.4)	25 (5.0)	0.198	0.49 (0.17–1.45)
		rs2276464	3′–UTR	GG	101 (59.8)	310 (61.9)		
				GC	64 (37.9)	165 (32.9)	0.367	1.18 (0.82–1.71)
				CC	4 (2.4)	22 (4.4)	0.289	0.56 (0.19–1.65)
XPG(ERCC5)	13q33	rs2094258	Promoter	GG	55 (32.5)	187 (37.3)		
				GA	92 (54.4)	229 (45.7)	0.116	1.36 (0.93–2.01)
				AA	22 (13.0)	75 (15.0)	0.965	1.01 (0.58–1.78)
		rs751402	Promoter	CC	70 (41.4)	232 (46.3)		
				CT	81 (47.9)	185 (36.9)	0.051	1.45 (1.00–2.11)
				TT	18 (10.7)	60 (12.0)	0.996	1.00 (0.55–1.81)

		rs2296147	Promoter	TT	113 (66.9)	319 (63.7)		
				TC	51 (30.2)	154 (30.7)	0.717	0.93 (0.64–1.37)
				CC	5 (3.0)	24 (4.8)	0.296	0.59 (0.22–1.59)
		rs1047768	Exon 2	TT	91 (53.8)	254 (50.7)		
				TC	65 (38.5)	200 (39.9)	0.618	0.91 (0.63–1.32)
				CC	12 (7.1)	44 (8.8)	0.445	0.77 (0.39–1.52)
		rs4150291	Intron 5	AA	15) 988.8)	418 (83.4)		
				AT	19 (11.2)	77 (15.4)	0.168	0.69 (0.40–1.17)
				TT	0 (0.0)	5 (1.0)	NA	NA
		rs2228959	Exon 8	CC	157 (92.9)	453 (90.4)		
				CA	12 (7.1)	43 (8.6)	0.516	0.80 (0.41–1.56)
				AA	0 (0.0)	4 (0.8)	NA	NA
		rs4150383	3′ near region	GG	147 (87.0)	433 (86.4)		
				GA	21 (12.4)	63 (12.6)	0.932	0.98 (0.58–1.66)
				AA	1 (0.6)	2 (0.4)	0.765	1.44 (0.13–16.07)
		rs873601	3′–UTR	GG	54 (32.0)	142 (28.3)		
				GA	87 (51.5)	231 (46.1)	0.966	0.99 (0.66–1.48)
				AA	28 (16.6)	126 (25.1)	**0.041**	**0.58 (0.35–0.98)**

Chr. Pos. = chromosomal position, meant the gene located in the chromosome.
Loc. = localisation, meant the SNP located in the relative location of the genes.
*the sort order was according to the SNP location in its genes from 5′ starting to 3′ ends.
†*P* value was calculated by adjusted age and sex.

Supplementary Table S3: Associations between genotype distributions of the positive *XPC* rs1870134 and clinicopathological parameters of hepatocellular cancer

Parameters	Genotype		*P*-value	GC + CC vs GG
	GC + CC	GG		OR (95% CI)
**Differentiation**				
Well	11 (27.5)	15 (23.4)		1
Moderate	20 (50.0)	35 (54.7)	0.995	1.00 (0.31-–3.25)
Poor	9 (22.5)	14 (21.9)	0.821	0.89 (0.33–2.40)
**Smoking**				
Ever Smoker	65 (33.3)	86 (30.6)		1
Never Smoker	130 (66.7)	195 (69.4)	0.308	1.25 (0.82–1.90)
**Drinking**				
Drinker	40 (20.5)	52 (18.5)		1
Nondrinker	154 (79.0)	229 (81.5)	0.45	1.20 (0.75–1.94)
**Family history**				
Yes	25 (21.2)	42 (19.7)		1
No	93 (78.8)	171 (80.3)	0.717	1.11 (0.63–1.94)
**HBV**				
Positive	79 (73.1)	164 (81.2)		1
Negative	29 (26.9)	38 (18.8)	0.185	0.68 (0.39–1.20)
**HCV**				
Positive	18 (29.0)	20 (21.1)		1
Negative	44 (71.0)	75 (78.9)	0.295	1.49 (0.71–3.13)

Supplementary Table S4: Primers for realtime PCR reaction for gene expression detection

Amplified region	Forward and Reverse primers (5′–3′)	Amplicon size	Annealing temperature
XPC mRNA[a]	F: TCCGAGATGTCACACAGAGG	102 bp	60°C
	R: TCTGGTATGGTCTCAAGGTCTC		
GAPDH mRNA[b]	F: TGCACCACCAACTGCTTAG	176 bp	60°C
	R: GGATGCAGGGATGATGTTC		

[a,]The primers used for XPC mRNA detection were according to Jana Slyskova et al [1].

[b,]The primers used for GAPDH mRNA detection were according to Poulin H et al [2].

[1] Jana Slyskova et al, Differences in nucleotide excision repair capacity between newly diagnosed colorectal cancer patients and healthy controls. Mutagenesis. 2012; 27:225–232.

[2] Poulin H et al, Labelle Y: The PLAGL1 gene is down-regulated in human extraskeletal myxoid chondrosarcoma tumors. Cancer Lett. 2005; 227:185–191.
